# Long Noncoding RNA *SNHG7* Is a Diagnostic and Prognostic Marker for Colon Adenocarcinoma

**DOI:** 10.3389/fonc.2022.893591

**Published:** 2022-06-07

**Authors:** Chengwei Jiang, Shanshan Qu, Tie Liu, Miao Hao

**Affiliations:** ^1^ Department of Pathology, China-Japan Union Hospital of Jilin University, Changchun, China; ^2^ Department of Pathology, China-The Second Hospital of Jilin University, Changchun, China; ^3^ Biobank, China-Japan Union Hospital of Jilin University, Changchun, China; ^4^ Scientific Research Center, China-Japan Union Hospital of Jilin University, Changchun, China

**Keywords:** SNHG7, bioinformatics, diagnosis, prognosis, colon adenocarcinoma

## Abstract

Numerous studies have shown that long noncoding RNAs (lncRNAs) play a critical role in the malignant progression of cancer. However, the potential involvement of lncRNAs in colon adenocarcinoma (COAD) remains unexplored. In this study, the expression of lncRNA *SNHG7* in colon cancer tissues and its correlation with clinical characteristics were analyzed based on data from The Cancer Genome Atlas (TCGA) database. *SNHG7* was found to be highly expressed in 17 types of cancer, including COAD. Next, TCGA data were further investigated to identify differentially expressed genes, and Gene Ontology and Kyoto Encyclopedia of Genes and Genomes analyses were performed. In addition, the relationship between *SNHG7* expression and clinical features were analyzed. *SNHG7* expression was found to be a potentially valuable indicator for COAD diagnosis and prognosis. Finally, gene set enrichment analysis showed that *SNHG7* may affect lupus erythematosus and reactome cellular senescence, possibly influencing the prognosis of patients with COAD. Altogether, these results suggest that *SNHG7* may be associated with the occurrence and development of COAD, having potential diagnostic and prognostic value.

## Introduction

Colon adenocarcinoma (COAD) is the second most lethal malignancy worldwide, which is currently treated surgically and/or using chemotherapy and radiotherapy (1). Although the overall survival (OS) rate has improved, invasion and metastasis remain the main death cause among patients with COAD (2). Extensive studies have shown that tumor biomarkers are highly sensitive and specific for diagnosing and monitoring tumors (3). Therefore, there is a critical need to identify new diagnostic or prognostic biomarkers and develop novel therapeutic strategies for COAD.

Long noncoding RNAs (lncRNAs) are a class of noncoding RNA transcripts that are over 200 nucleotides in length. The dysregulation of lncRNAs is closely related to various major diseases, including cancer (4). Many studies have shown that cancer- associated lncRNAs are involved in the regulation of tumor proliferation, invasion, and metastasis; thus, are considered to be a class of potential candidate biomarkers for cancer diagnosis and therapy (5). For example, the lncRNA *HOTAIR* is an oncogene that is upregulated in breast cancer tissues and is closely related to poor prognosis and tumor metastasis (6). *MALAT1* (metastasis-associated lung adenocarcinoma transcript 1) is a lncRNA that was originally found to be abundantly expressed in metastatic carcinoma cells and to be significantly upregulated in various types of cancer, such as breast cancer (7) and non-small cell lung cancer (8), being suggested as a prognostic biomarker and potential therapeutic target for metastatic cancers (9). *H19* is an estrogen-regulated lncRNA transcript whose aberrant expression is closely associated with cell proliferation and migration in a variety of cancers, such as gastric, gallbladder, and pancreatic cancers (10). Although lncRNAs have been broadly recognized to play important regulatory roles in human cancers, few have been demonstrated to function in COAD, and most of their mechanisms are largely unknown.

Small nucleolar RNA host genes (SNHGs) are newly recognized lncRNAs that have oncogenic roles in various cancers (11). Members of the SNHG family have been shown to regulate cellular proliferation, apoptosis, invasion, and migration in multiple cancers (12). LncRNA *SNHG7* is closely related to the occurrence, development, and carcinogenesis potential of numerous cancers, including lung, gastric and cervical cancer, as well as renal cell carcinoma and hepatocellular carcinoma (13–15). Nevertheless, few reports have explored the impact of *SNHG7* on COAD. This study aimed to investigate the relationship between the expression of *SNHG7* and the prognosis of COAD using bioinformatics tools.

## Materials and Methods

### Data Collection

RNA sequencing data from 521 COAD samples and associated clinical information were obtained from The Cancer Genome Atlas (TCGA) database (https://portal.gdc.cancer.gov/). Another RNA sequencing data of 698 COAD samples and clinical information were also included for validation. RNA sequencing data were converted from fragments per kilobyte per million (FPKM) to the transcripts per million reads (TPM) format, and compared according to the corresponding clinicopathological information. As all data collected was publicly available, informed consent and ethical approval were not necessary to obtain.

### Clinical Significance and Correlation of *SNHG7* Expression in COAD Patients

To clarify the association between *SNHG7* expression and clinical features of COAD, Wilcoxon signed-rank test and logistic regression were performed. The detailed clinicopathological characteristics of the patients with COAD are listed in **Table 1**.

**Table 1:** The relationships between SNHG7 expression and clinicopathological features in COAD patients.

Characteristic	No.	Low expression of SNHG7	High expression of SNHG7	*p*
Gender				0.314
Female	226	119 (24.9%)	107 (22.4%)	
Male	252	120 (25.1%)	132 (27.6%)	
Age				0.780
≤65	194	99 (20.7%)	95 (19.9%)	
>65	284	140 (29.3%)	144 (30.1%)	
Race				0.046*
Asian	11	2 (0.7%)	9 (2.9%)	
Black or African American	63	36 (11.8%)	27 (8.8%)	
White	232	131 (42.8%)	101 (33%)	
T stage				0.062
T1	11	4 (0.8%)	7 (1.5%)	
T2	83	45 (9.4%)	38 (8%)	
T3	323	168 (35.2%)	155 (32.5%)	
T4	60	21 (4.4%)	39 (8.2%)	
N stage				0.249
N0	284	147 (30.8%)	137 (28.7%)	
N1	108	56 (11.7%)	52 (10.9%)	
N2	86	36 (7.5%)	50 (10.5%)	
Residual tumor				0.045*
R0	346	173 (46.3%)	173 (46.3%)	
R1	4	2 (0.5%)	2 (0.5%)	
R2	24	6 (1.6%)	18 (4.8%)	
Perineural invasion				0.630
NO	135	75 (41.4%)	60 (33.1%)	
YES	46	23 (12.7%)	23 (12.7%)	
Lymphatic invasion				0.388
NO	266	144 (33.2%)	122 (28.1%)	
YES	168	83 (19.1%)	85 (19.6%)	
OS event				0.026*
Alive	375	198 (41.4%)	177 (37%)	
Dead	103	41 (8.6%)	62 (13%)	
DSS event				0.008**
Alive	398	211 (45.7%)	187 (40.5%)	
Dead	64	22 (4.8%)	42 (9.1%)	

*P < 0.05, **P < 0.01.

To assess the predictive potential of *SNHG7* for COAD diagnosis, *SNHG7* expression in COAD and normal tissues was compared using receiver operating characteristic (ROC) analysis. COAD and corresponding normal tissue data were obtained from the TCGA database. The analysis was performed using the R package “pROC” (version1.17.0.1), and the visualization was achieved using “ggplot2” (version 3.3.3).

Kaplan–Meier analysis, and univariate and multivariate Cox regression analyses were used for prognosis analysis. Nomograms were created using the R packages “rms” (version 6.2-0) and “survival” (version 3.2-10). R (v3.6.3; R Foundation for Statistical Computing, Vienna, Austria) was used to conduct all statistical studies, with *p*-values below 0.05 deemed significant.

### Screening of Differentially Expressed Genes (DEGs), and Gene Ontology (GO) and Kyoto Encyclopedia of Genes and Genomes (KEGG) Analyses

COAD gene expression data in the HTSeq-TPM format were obtained from TCGA for analysis. *SNHG7* coexpressed genes were screened using Pearson correlation coefficients (|*r*| > 0.4 and *p* < 0.001). To explore the possible biological functions and signaling pathways affected by *SNHG7*, the R package “cluster Profiler” was used to perform GO and KEGG analyses of coexpressed genes, with *p* < 0.05 deemed statistically significant. GO analysis included biological processes (BP), cell composition (CC), and molecular function (MF).

### Gene Set Enrichment Analysis (GSEA)

GSEA is a computational method used to determine whether an *a priori* defined gene set exhibits statistically significant and consistent differences between two biological states (16). In the present study, we elucidated the survival differences between groups with high and low *SNHG7* expression using GSEA. Gene set permutations were performed 1,000 times for each analysis. The expression of *SNHG7* was used as the phenotypic label. The nominal *p*-value and normalized enrichment score (NES) were used to identify the pathways enriched for each phenotype.

## Results

### Expression Profiles of *SNHG7* in Pan-Cancer Datasets

**Figure 1:**
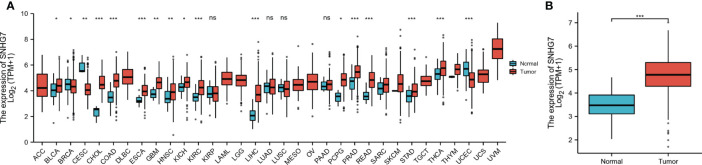
Expression level of SNHG7 in different tumors and in COAD. **(A)** The comparison of SNHG7 expression between normal and tumor tissue in different types of cancers based on TCGA database. **(B)** SNHG7 expression is significantly higher in COAD (n = 480) than normal tissue (n = 41). ns, *P* ≥ 0.05, *P < 0.05, **P < 0.01, ***P < 0.001.

Based on TCGA data analysis, we found that *SNHG7* was upregulated in 17 of the 33 cancer types investigated, including cholangiocarcinoma (CHOL), prostate adenocarcinoma (PRAD), and thyroid carcinoma (THCA) (**Figure 1A**). Further analysis showed that *SNHG7* expression was much higher in patients with COAD than that in normal tissues (p < 0.001, **Figure 1B**). These findings indicate that *SNHG7* may play a significant regulatory role in the progression of COAD.

### Clinical Correlation Analyses

Clinical information, including sex, age, race, T stage, N stage, residual tumor, perineural invasion, lymphatic invasion, OS, and disease-specific survival (DSS) (**Table 1**), for 521 COAD patients was obtained from TCGA database. *SNHG7* expression was not only significantly correlated with race (p < 0.05) and residual tumor (p < 0.05), but was also closely correlated with OS (p < 0.05) and DSS (p < 0.01). No correlation was observed between *SNHG7* expression and the other clinicopathological characteristics.

### Diagnostic Value of *SNHG7* in COAD Patients

**Figure 2:**
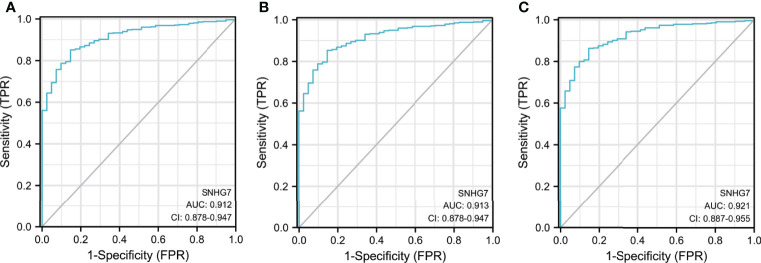
The diagnostic value of SNHG7 in COAD patients. **(A)** ROC curve of SNHG7 in diagnosing of COAD patients. **(B)** ROC curve of SNHG7 in T staging of COAD patients. **(C)** ROC curve of SNHG7 in M staging of COAD patients and 0.921 (95% CI: 0.887-0.955), respectively (Figure 2B&C).

ROC curves were used to evaluate the potential of *SNHG7* expression to identify patients with COAD. *SNHG7* expression had high sensitivity and specificity for COAD diagnosis, with an area under the curve (AUC) of 0.912 (95% confidence interval [CI], 0.878–0.947) (**Figure 2A**). Further analysis showed that *SNHG7* expression could diagnose T and N stages, with AUC values of 0.913 (95% CI: 0.878–0.947) and 0.921 (95% CI: 0.887–0.955), respectively (**Figures 2B, C**). Taken together, these results suggested that *SNHG7* expression could represent a valuable tool to diagnosis COAD.

### Relationship Between *SNHG7* Expression and Prognosis of COAD Patients

**Figure 3:**
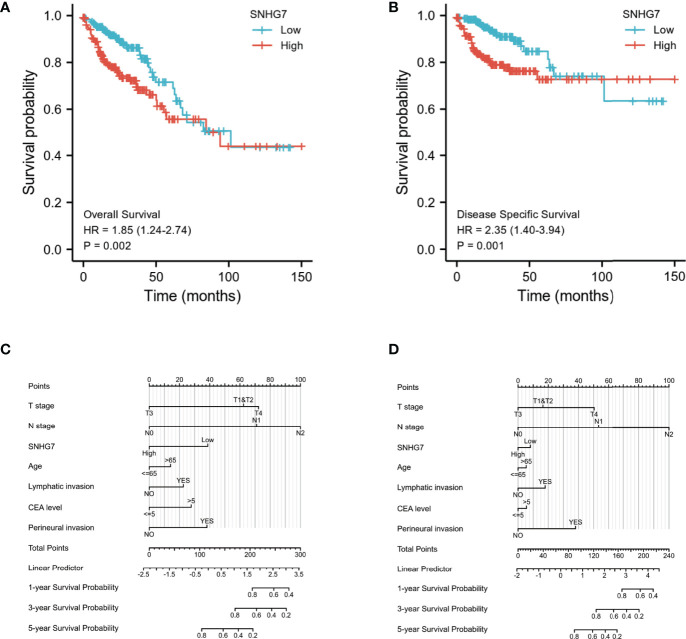
Relationship between SNHG7 expression and survival rate in COAD analyzed by Kaplan–Meier **(A, B)**. Construction and validation of nomograms based on SNHG7 expression **(C, D)**. **(A)** The relationship between overall survival and SNHG7 expression. **(B)** The relationship between disease-specific survival and SNHG7 expression. **(C)** Nomograms constructed to establish SNHG7 expression-based risk scoring models for 1-, 3-, and 5-year overall survival. **(D)** Nomograms constructed to establish SNHG7 expression-based risk scoring models for 1-, 3-, and 5-year disease-specific survival.

Kaplan–Meier analysis was used to confirm whether *SNHG7* expression could be used for the prediction of clinical outcomes among patient with COAD. Overall, high *SNHG7* expression was associated to shorter OS (hazard ratio [HR]: 1.85, *p* = 0.002) and DSS (HR: 2.35, *p* = 0.001) in COAD patients (**Figures 3A, B**). To determine whether *SNHG7* expression had a predictive value for clinical outcomes, we also performed univariate and multivariate Cox regression analyses. As shown in **Table 2**, *SNHG7* expression (HR: 1.847, 95% CI: 1.244–2.741, *p* = 0.002) was an independent risk factor for OS. Age, T stage, pathological stage, lymphatic invasion, and carcinoembryonic antigen (CEA) level also showed prognostic advantages for clinical outcomes. In addition, the N stage showed prognostic advantages in both univariate (HR: 4.051, 95% CI: 2.593–6.329, *p* < 0.001) and multivariate (HR: 6.048, 95% CI: 1.006–36.361, *p* = 0.049) Cox regression analyses. Furthermore, *SNHG7* expression, T stage, N stage, pathological stage, perineural invasion, lymphatic invasion, and CEA level were all independent risk factors for DSS (**Table 3**). N stage (HR: 2.933, 95% CI: 0.218–39.407, *p* = 0.021) was an independent risk factor for DSS.

**Table 2:** Univariate and multivariate Cox proportional hazards analysis of SNHG7 expression and OS (overall survival) for patients with COAD in the validation cohort.

Characteristics	Total (N)	Univariate analysis			Multivariate analysis	
		Hazard ratio (95% CI)	P value		Hazard ratio (95% CI)	P value
Gender	477					
Female	226	Reference				
Male	251	1.101 (0.746-1.625)	0.627			
Age	477					
<=65	194	Reference				
>65	283	1.610 (1.052-2.463)	0.028*		1.296 (0.488-3.444)	0.603
T stage	476					
T1 & T2	94	Reference				
T3	322	2.576 (1.183-5.612)	0.017*		0.301 (0.073-1.238)	0.096
T4	60	7.021 (2.993-16.473)	<0.001**		1.367 (0.283-6.614)	0.697
N stage	477					
N0	283	Reference				
N1	108	1.681 (1.019-2.771)	0.042*		3.687 (0.922-14.754)	0.065
N2	86	4.051 (2.593-6.329)	<0.001**		6.048 (1.006-36.361)	0.049*
Pathologic stage	466					
Stage I & Stage II	267	Reference				
Stage III & Stage IV	199	2.947 (1.942-4.471)	<0.001**			
Perineural invasion	181					
NO	135	Reference				
YES	46	1.940 (0.982-3.832)	0.056		2.249 (0.747-6.770)	0.150
Lymphatic invasion	433					
NO	265	Reference				
YES	168	2.450 (1.614-3.720)	<0.001**		1.454 (0.472-4.476)	0.514
CEA level	302					
<=5	195	Reference				
>5	107	3.128 (1.788-5.471)	<0.001**		1.822 (0.663-5.009)	0.245
SNHG7	477					
Low	239	Reference				
High	238	1.847 (1.244-2.741)	0.002**		0.438 (0.151-1.267)	0.128

CI, confidence interval; HR, hazard ratio. *P < 0.05, **P < 0.01.

**Table 3:** Univariate and multivariate Cox proportional hazards analysis of SNHG7 expression and DSS (Disease Specific Survival) for patients with COAD in the validation cohort.

Characteristics	Total (N)	Univariate analysis			Multivariate analysis	
		Hazard ratio (95% CI)	P value		Hazard ratio (95% CI)	P value
Gender	461					
Female	220	Reference				
Male	241	1.142 (0.697-1.871)	0.599			
Age	461					
<=65	191	Reference				
>65	270	1.165 (0.702-1.933)	0.555			
T stage	460					
T1&T2	93	Reference				
T3	307	5.984 (1.447-24.751)	0.014*		0.634 (0.053-7.643)	0.720
T4	60	20.180 (4.693-86.773)	<0.001**		2.933 (0.218-39.407)	0.417
N stage	461					
N0	275	Reference				
N1	105	2.601 (1.353-5.000)	0.004**		4.141 (0.586-29.247)	0.154
N2	81	6.357 (3.512-11.504)	<0.001**		15.636 (1.525-160.294)	0.021*
Pathologic stage	451					
Stage I&Stage II	259	Reference				
Stage III&Stage IV	192	6.085 (3.235-11.447)	<0.001**			
Perineural invasion	180					
NO	134	Reference				
YES	46	2.977 (1.325-6.686)	0.008**		3.136 (0.800-12.298)	0.101
Lymphatic invasion	422					
NO	255	Reference				
YES	167	4.133 (2.361-7.235)	<0.001**		1.560 (0.321-7.589)	0.582
CEA level	301					
<=5	194	Reference				
>5	107	3.018 (1.543-5.901)	0.001**		1.273 (0.350-4.633)	0.714
SNHG7	461					
Low	233	Reference				
High	228	2.353 (1.404-3.944)	0.001**		0.736 (0.194-2.798)	0.653

CI, confidence interval; HR, hazard ratio. *P < 0.05, **P < 0.01.

Based on the significant prognostic factors identified in the Cox regression analysis, prognostic nomograms were designed. Age, pathological stage, perineural or lymphatic invasion, and *SNHG7* expression were included in the nomogram to predict OS (C-index = 0.836) (**Figure 3C**) and DSS (C-index = 0.875) (**Figure 3D**). These results indicated that *SNHG7* expression was not only significantly upregulated in COAD but also had prognostic value, suggesting that *SNHG7* has important regulatory functions in this type of cancer.

### Coexpressed Genes of *SNHG7* and Functional Annotation of SNHG7-Associated DEGs in COAD

**Figure 4:**
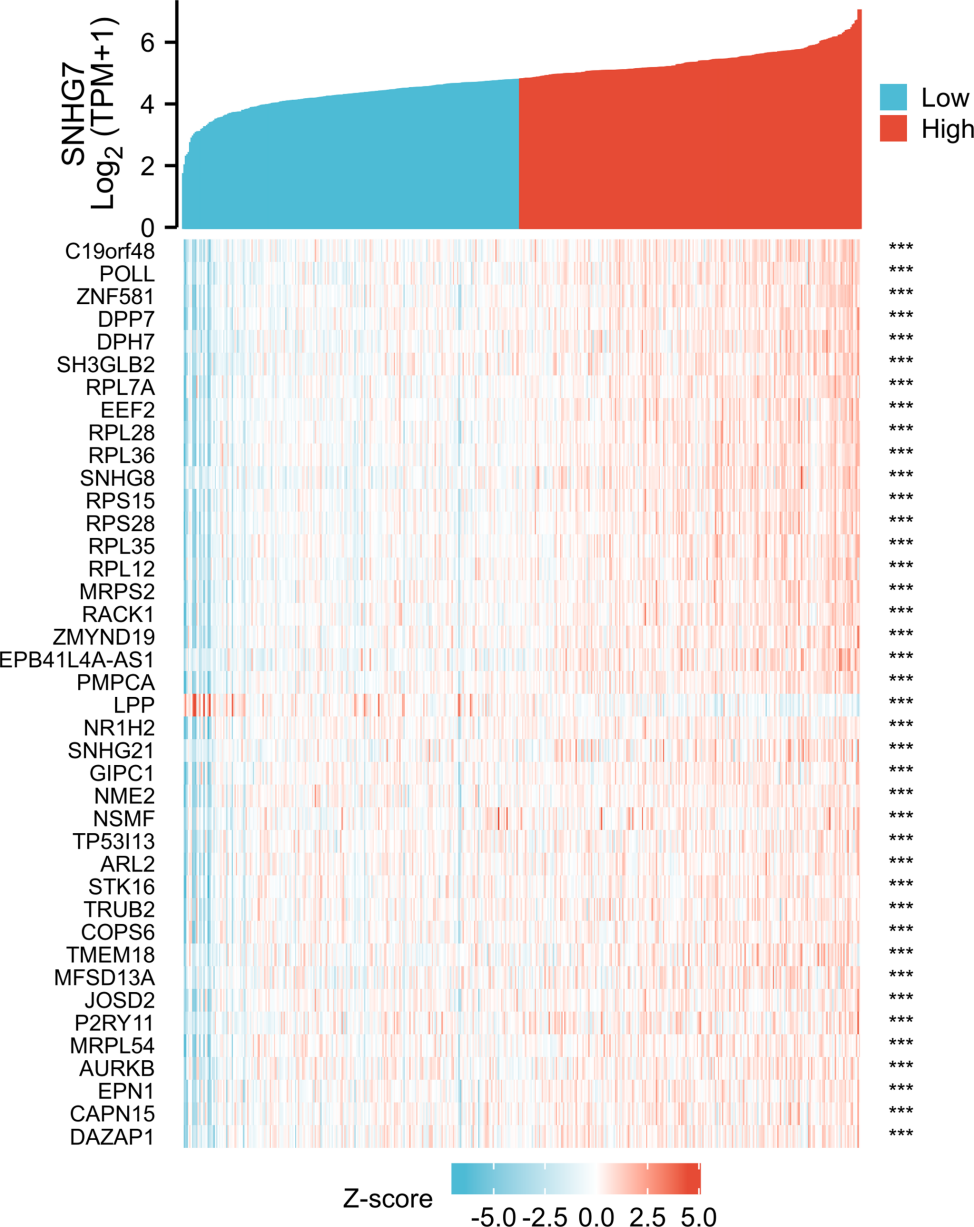
The top 20 genes with positive and negative co-expression of SNHG7 in TCGA database according to Heatmap in COAD. ***P<0.001.

To screen for coexpressed genes of *SNHG7*, Pearson correlation coefficients were set as |*r*| > 0.4 and *p* < 0.001. The top 20 positively and negatively correlated coexpressed genes of *SNHG7* are displayed in the form of a heatmap (**Figure 4**).

**Figure 5:**
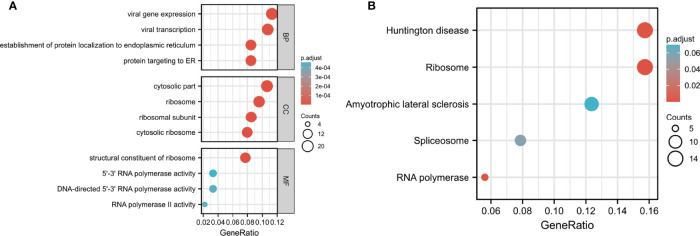
GO and KEGG pathway analyses of DEGs in COAD. **(A)** Biological process (BF), cell components (CC), and molecular function (MF) enrichment analyses of DEGs. **(B)** KEGG pathway analysis of DEGs.

Next, we performed GO and KEGG analysis of *SNHG7*- associated DEGs in COAD. GO analysis demonstrated that genes of GO-BP terms were significantly enriched in viral gene expression, viral transcription, establishment of protein localization to the endoplasmic reticulum (ER), and protein targeting to the ER. For GO-CC terms, the genes were mainly located in the cytosolic part, ribosome, ribosomal subunit, and the cytosolic ribosome. In GO-MF analysis, genes were enriched in the structural constituent of ribosomes, 5′–3′ RNA polymerase activity, DNA-directed 5′– 3′ RNA polymerase activity, and RNA polymerase II activity (**Table 4**; **Figure 5A**). As shown in **Figure 5B**; **Table 5**, KEGG pathway analysis indicated that the top pathways were mainly associated with Huntington’s disease, ribosome, amyotrophic lateral sclerosis, spliceosome, and RNA polymerases.

**Table 4:** GO analysis of SNHG7 co-expression genes.

ONTOLOGY	ID	Description	GeneRatio	pvalue	p.adjust
BP	GO:0019080	viral gene expression	20/177	1.75e-15	3.72e-12
BP	GO:0019083	viral transcription	19/177	5.81e-15	6.16e-12
BP	GO:0045047	protein targeting to ER	15/177	3.82e-13	2.70e-10
BP	GO:0072599	establishment of protein localization to endoplasmic reticulum	15/177	6.30e-13	3.33e-10
BP	GO:0006614	SRP-dependent cotranslational protein targeting to membrane	14/177	1.22e-12	5.18e-10
CC	GO:0022626	cytosolic ribosome	15/188	1.95e-13	4.45e-11
CC	GO:0044445	cytosolic part	20/188	2.84e-13	4.45e-11
CC	GO:0044391	ribosomal subunit	16/188	4.16e-11	4.34e-09
CC	GO:0005840	ribosome	18/188	1.33e-10	1.04e-08
CC	GO:0022625	cytosolic large ribosomal subunit	9/188	8.17e-09	5.11e-07
MF	GO:0003735	structural constituent of ribosome	14/181	2.12e-08	7.88e-06
MF	GO:0001055	RNA polymerase II activity	4/181	3.30e-06	4.36e-04
MF	GO:0003899	DNA-directed 5’-3’ RNA polymerase activity	6/181	3.52e-06	4.36e-04
MF	GO:0034062	5’-3’ RNA polymerase activity	6/181	6.16e-06	4.58e-04
MF	GO:0097747	RNA polymerase activity	6/181	6.16e-06	4.58e-04

**Table 5:** KEGG analysis of SNHG7 co-expression genes.

ID	Description	GeneRatio	pvalue	p.adjust
hsa03010	Ribosome	14/89	1.45e-09	2.44e-07
hsa05016	Huntington disease	14/89	5.47e-06	4.60e-04
hsa03020	RNA polymerase	5/89	1.97e-05	0.001
hsa03040	Spliceosome	7/89	0.001	0.056
hsa05014	Amyotrophic lateral sclerosis	11/89	0.002	0.070

### GSEA of *SNHG7*

**Figure 6:**
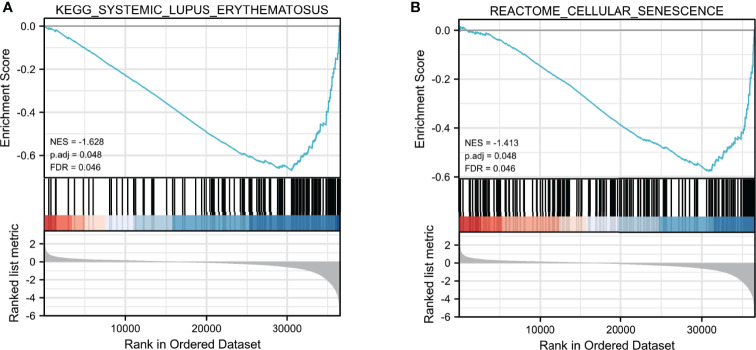
Enrichment plots from GSEA analysis. GSEA analysis showed that Lupus erythematosus **(A)** and Reactome cellular senescence **(B)** were differentially enriched between high- and low-SNHG7 expression groups.

To further clarify biological functions of *SNHG7* in COAD, GSEA enrichment analysis was performed on the high and low expression datasets of *SNHG7*. Significant differences (false discovery rate < 0.25, adjusted *p* < 0.05) were observed in the enrichment of the MSigDB Collection (c2.cp.v7.2.symbols.gmt). The most markedly enriched signaling pathways were screened based on their NES (**Figures 6A, B**). The results illustrated that lupus erythematosus (NES = −1.628, adjusted *p* = 0.048) and reactome cellular senescence (NES = −1.413, adjusted *p* = 0.048) were mainly enriched in the highly expressed *SNHG7* phenotype.

### Validation of Differential Expression, Prognostic and Diagnostic Value of *SNHG7* in Other Independent Cohorts of COAD

**Figure 7:**
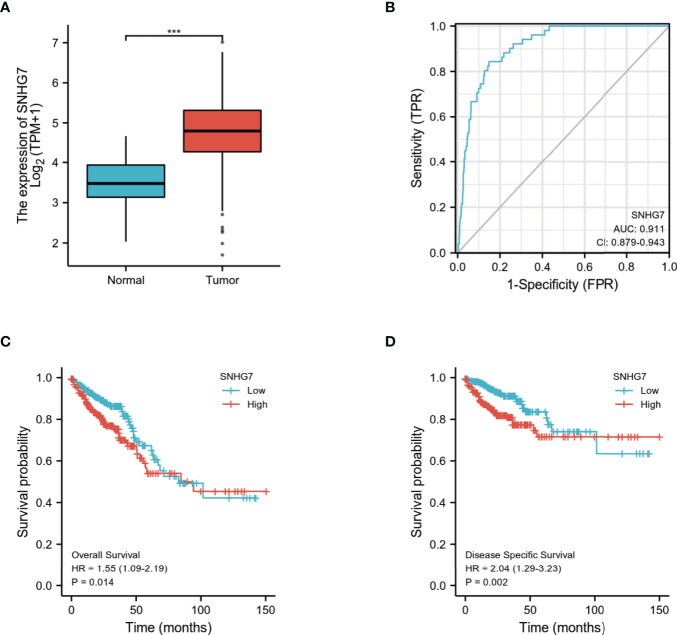
Validation of differential expression, prognostic and diagnostic value of SNHG7 in COAD (n=698) **(A)** SNHG7 expression is significantly higher in COAD (n = 647) than normal tissue (n = 51). ***P < 0.001. **(B)** ROC curve of SNHG7 in diagnosing of COAD patients. **(C)** The relationship between overall survival and SNHG7 expression. **(D)** The relationship between disease-specific survival and SNHG7 expression.

To validate the prognostic robustness and clinical reproducibility of *SNHG7*, an independent cohort available at TCGA database, comprising 698 samples, was also analyzed. As shown in **Figure 7A**, *SNHG7* expression was significantly upregulated in the tumor group as compared with normal tissues (*p* < 0.001). Similarly, ROC curve analysis also indicated that *SNHG7* had a very high diagnostic value in COAD (AUC = 0.911, 95% CI: 0.879−0.943) (**Figure 7B**). Analysis of the OS (**Figure 7C**; HR: 1.55, *p* = 0.014) and DSS (**Figure 7D**; HR: 2.04, *p* = 0.002) of these patients further suggested that high *SNHG7* expression was correlated with poor prognosis in COAD. These results were consistent with the conclusions of *SNHG7* in cohort of 521 samples, indicating that the diagnostic and prognostic value of *SNHG7* in COAD is credible and reproducible.

## Discussion

Currently, the 5-year survival rate of early COAD exceeds 70−90%; nonetheless, the curative effect for advanced COAD is still not ideal, which is mainly due to its high recurrence and metastasis rate (17, 18). Therefore, the development of biomarkers aiding early differential diagnosis and predicting COAD progression is of major importance both for research and therapeutic evolution (19). It has been established that lncRNAs may be potential diagnostic and/or prognostic markers for clinical applications. In particular, many lncRNA biomarkers were reported for colorectal cancer (20, 21).

*SNHG7*, which is a member of the *SNHG* family, is differentially expressed in various malignant tumors (13–15). Noteworthy, recent studies revealed that *SNHG7* has a regulatory role in colorectal cancer. For example, *SNHG7* is an oncogenic biomarker in COAD, and it interacts with miR-193b (22) and positively regulates GALNT1 levels through sponging miR-216b in colorectal cancer (23). Moreover, *SNHG7* and FAIM2 are upregulated in colorectal cancer tissues compared with normal adjacent tissues (24). However, the possible clinical significance and prognostic/diagnostic value of *SNHG7*

in COAD remain unclear. Therefore, the development of new and effective biomarkers for the prognosis and early diagnosis of COAD would be beneficial to enhance the treatment and prognosis of patients.

To gain a comprehensive understanding of the role of *SNHG7* in COAD, we first identified the differential expression of *SNHG7* using publicly available pan-cancer data. We confirmed that *SNHG7* is differentially expressed in multiple tumors; in particular *SNHG7* expression was significantly upregulated in COAD compared with other tumors. These findings suggest that *SNHG7* differential expression may be tissue- specific and it may have an important regulatory role in COAD.

To further test our hypothesis, we analyzed the clinical relationship of *SNHG7* in COAD by univariate and multivariate Cox regression analyses. We discovered a strong associated between *SNHG7* expression and race, residual tumor, OS, and DSS of COAD patients, with *SNHG7* expression appearing to be higher in patients with certain characteristics, such as specific race and with residual tumor. Moreover, we demonstrated that high *SNHG7* expression was associated with significantly shorter OS and DSS in COAD patients, but was also an independent risk factor for OS and DSS.

Histopathological characteristics have been implicated as prognostic predictors, such as tumor stages, perineural invasion, and lymphatic invasion (25). Our results also confirmed that these three prognostic predictors were closely related to poor prognosis for OS and DSS in COAD patient with high *SNHG7* expression. Noteworthily, in line with our *SNHG7-*based predicted outcome, univariate Cox regression analysis showed that high CEA levels, which are an independent prognostic factor and can be used for

TNM staging of COAD, reflected poor prognosis in COAD (26). Hence, the remarkable predictive ability of SNHG7 expression suggests its potential as a prognostic biomarker of poor survival in COAD.

In addition, we explored the potential functions and underlying mechanisms of action of *SNHG7* in COAD. GO and KEGG analyses revealed that both ribosome and RNA polymerase were closely related to *SNHG7* based on the functional annotation of *SNHG7*-related DEGs. These results also indicated that *SNHG7* expression is closely associated with COAD.

The accuracy of a diagnostic tool is based on the area under the ROC curve; the closer the area under the ROC curve is to 1, the better the diagnostic potential of the tool (27). Our results consistently revealed that high *SNHG7* expression led to advanced COAD, indicating that *SNHG7* expression had high sensitivity and specificity for COAD diagnosis. Assessment of an independent COAD cohort further confirmed the differential expression of *SNHG7*, and its diagnostic and prognostic value in COAD, indicating that *SNHG7* is reliable and reproducible as a prognostic and diagnostic biomarker of COAD.

## Conclusions

In conclusion, this study demonstrated that COAD is associated with high *SNHG7* expression and that *SNHG7* is a reliable biomarker for the diagnosis and prognosis of COAD. Hence, these findings may represent new foundations for the development of enhanced diagnostic and prognostic strategies for COAD.

## Data Availability Statement

The datasets presented in this study can be found in online repositories. The names of the repository/repositories and accession number(s) can be found in the article/**Supplementary Material**.

## Ethics Statement

The studies involving human participants were reviewed and approved by The TCGA database. The patients/participants provided their written informed consent to participate in this study. Written informed consent was obtained from the individual(s) for the publication of any potentially identifiable images or data included in this article.

## Author Contributions

MH: design. CJ and SQ: acquisition of data. CJ, SQ, and TL: analysis and interpretation of data. CJ and MH: writing, review, and/or revision of the manuscript. SQ and MH: study supervision. All authors contributed to the article and approved the submitted version.

## Funding

This research was supported by a grant from Department of Science and Technology of Jilin Province.

## Conflict of Interest

The authors declare that the research was conducted in the absence of any commercial or financial relationships that could be construed as a potential conflict of interest.

## Publisher’s Note

All claims expressed in this article are solely those of the authors and do not necessarily represent those of their affiliated organizations, or those of the publisher, the editors and the reviewers. Any product that may be evaluated in this article, or claim that may be made by its manufacturer, is not guaranteed or endorsed by the publisher.
