# Supplementary material for: Long Noncoding RNA SNHG7 Is a Diagnostic and Prognostic Marker for Colon Adenocarcinoma
Source: Front Oncol. 2022 Jun 7;12:893591. doi: 10.3389/fonc.2022.893591 (PMC9209656; doi:10.3389/fonc.2022.893591)
Supplement: Supplementary file 3 [file Table_3.docx]

| ONTOLOGY | ID | Description | GeneRatio | BgRatio | pvalue | p.adjust | qvalue |
| --- | --- | --- | --- | --- | --- | --- | --- |
| BP | GO:0019080 | viral gene expression | 20/177 | 191/18670 | 1.75e-15 | 3.72e-12 | 3.48e-12 |
| BP | GO:0019083 | viral transcription | 19/177 | 177/18670 | 5.81e-15 | 6.16e-12 | 5.76e-12 |
| BP | GO:0045047 | protein targeting to ER | 15/177 | 118/18670 | 3.82e-13 | 2.70e-10 | 2.53e-10 |
| BP | GO:0072599 | establishment of protein localization to endoplasmic reticulum | 15/177 | 122/18670 | 6.30e-13 | 3.33e-10 | 3.12e-10 |
| BP | GO:0006614 | SRP-dependent cotranslational protein targeting to membrane | 14/177 | 105/18670 | 1.22e-12 | 5.18e-10 | 4.85e-10 |
| CC | GO:0022626 | cytosolic ribosome | 15/188 | 112/19717 | 1.95e-13 | 4.45e-11 | 3.59e-11 |
| CC | GO:0044445 | cytosolic part | 20/188 | 247/19717 | 2.84e-13 | 4.45e-11 | 3.59e-11 |
| CC | GO:0044391 | ribosomal subunit | 16/188 | 190/19717 | 4.16e-11 | 4.34e-09 | 3.51e-09 |
| CC | GO:0005840 | ribosome | 18/188 | 272/19717 | 1.33e-10 | 1.04e-08 | 8.40e-09 |
| CC | GO:0022625 | cytosolic large ribosomal subunit | 9/188 | 63/19717 | 8.17e-09 | 5.11e-07 | 4.13e-07 |
| MF | GO:0003735 | structural constituent of ribosome | 14/181 | 202/17697 | 2.12e-08 | 7.88e-06 | 7.47e-06 |
| MF | GO:0001055 | RNA polymerase II activity | 4/181 | 11/17697 | 3.30e-06 | 4.36e-04 | 4.14e-04 |
| MF | GO:0003899 | DNA-directed 5'-3' RNA polymerase activity | 6/181 | 41/17697 | 3.52e-06 | 4.36e-04 | 4.14e-04 |
| MF | GO:0034062 | 5'-3' RNA polymerase activity | 6/181 | 45/17697 | 6.16e-06 | 4.58e-04 | 4.35e-04 |
| MF | GO:0097747 | RNA polymerase activity | 6/181 | 45/17697 | 6.16e-06 | 4.58e-04 | 4.35e-04 |
