# Supplementary material for: Long Noncoding RNA SNHG7 Is a Diagnostic and Prognostic Marker for Colon Adenocarcinoma
Source: Front Oncol. 2022 Jun 7;12:893591. doi: 10.3389/fonc.2022.893591 (PMC9209656; doi:10.3389/fonc.2022.893591)
Supplement: Supplementary file 5 [file Table_5.docx]

| Ontology | ID | Description | GeneRatio | BgRatio | pvalue | p.adjust | qvalue |
| --- | --- | --- | --- | --- | --- | --- | --- |
| KEGG | hsa03010 | Ribosome | 14/89 | 158/8076 | 1.45e-09 | 2.44e-07 | 2.37e-07 |
| KEGG | hsa05016 | Huntington disease | 14/89 | 306/8076 | 5.47e-06 | 4.60e-04 | 4.46e-04 |
| KEGG | hsa03020 | RNA polymerase | 5/89 | 31/8076 | 1.97e-05 | 0.001 | 0.001 |
| KEGG | hsa03040 | Spliceosome | 7/89 | 151/8076 | 0.001 | 0.056 | 0.054 |
| KEGG | hsa05014 | Amyotrophic lateral sclerosis | 11/89 | 364/8076 | 0.002 | 0.070 | 0.068 |
